# Hotspots of Community Change: Temporal Dynamics Are Spatially Variable in Understory Plant Composition of a California Oak Woodland

**DOI:** 10.1371/journal.pone.0133501

**Published:** 2015-07-29

**Authors:** Erica N. Spotswood, James W. Bartolome, Barbara Allen-Diaz

**Affiliations:** 1 Department of Environmental Science, Policy and Management, University of California, Berkeley, California, United States of America; 2 Department of Agriculture and Natural Resources, University of California, Oakland, California, United States of America; Estacion Experimental de Zonas Aridas - CSIC, SPAIN

## Abstract

Community response to external drivers such climate and disturbance can lead to fluctuations in community composition, or to directional change. Temporal dynamics can be influenced by a combination of drivers operating at multiple spatial scales, including external landscape scale drivers, local abiotic conditions, and local species pools. We hypothesized that spatial variation in these factors can create heterogeneity in temporal dynamics within landscapes. We used understory plant species composition from an 11 year dataset from a California oak woodland to compare plots where disturbance was experimentally manipulated with the removal of livestock grazing and a prescribed burn. We quantified three properties of temporal variation: compositional change (reflecting the appearance and disappearance of species), temporal fluctuation, and directional change. Directional change was related most strongly to disturbance type, and was highest at plots where grazing was removed during the study. Temporal fluctuations, compositional change, and directional change were all related to intrinsic abiotic factors, suggesting that some locations are more responsive to external drivers than others. Temporal fluctuations and compositional change were linked to local functional composition, indicating that environmental filters can create subsets of the local species pool that do not respond in the same way to external drivers. Temporal dynamics are often assumed to be relatively static at the landscape scale, provided disturbance and climate are continuous. This study shows that local and landscape scale factors jointly influence temporal dynamics creating hotspots that are particularly responsive to climate and disturbance. Thus, adequate predictions of response to disturbance or to changing climate will only be achieved by considering how factors at multiple spatial scales influence community resilience and recovery.

## Introduction

Understanding why some communities can show resilience to external drivers, while others shift dramatically with little recovery, lies at the heart of a wide range of ecological questions including response to disturbance and climate change [1,2,3,4]. While external drivers can have direct effects on community structure, they can also influence species interactions causing strong indirect effects [5,6,7,8]. Moreover, these effects likely vary in space, because local abiotic conditions can modify the impact of external drivers as well as species interactions [9,10,11]. Much of the work exploring temporal dynamics has focused on effects at a local level, where dynamics of a particular site are followed through time [12,13,14]. However, due to complex interactions between external drivers, species composition and site characteristics, these dynamics are likely to be influenced by conditions at multiple spatial scales [15]. Just as there are hotspots of environmental functioning [16,17], spatial variation in drivers may create areas on the landscape that are much more responsive to temporal change compared to others [18]. Uncovering the conditions under which these hotspots occur would greatly improve our ability to predict recovery after a disturbance, active restoration, and increasing variability in climate; all of which depend on adequate understanding of how temporal dynamics may be influenced by local conditions.

**Fig 1:**
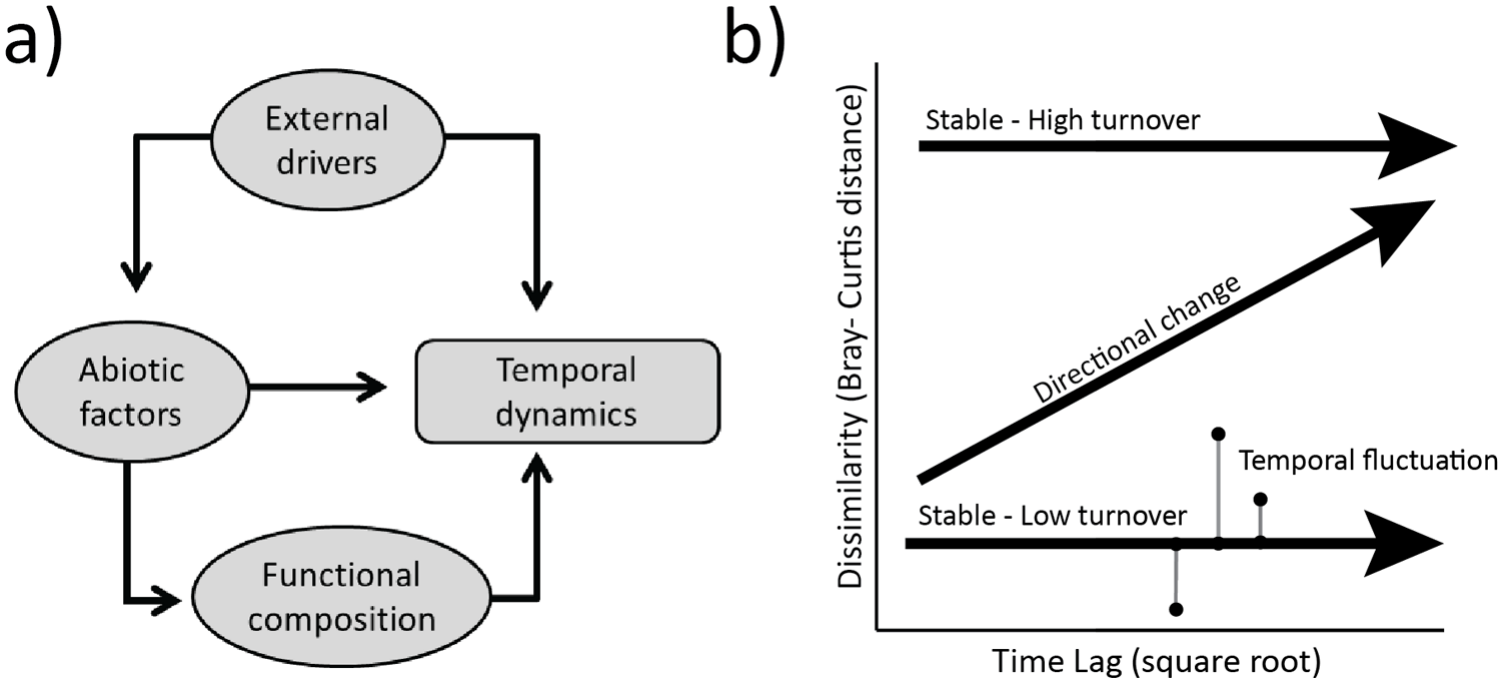
Conceptual framework for temporal dynamics. In a) a conceptual framework shows how landscape and local factors can affect temporal dynamics. Landscape scale external drivers can influence temporal dynamics directly, or can interact with local scale abiotic factors, creating spatial variation in temporal dynamics. Furthermore, abiotic factors can influence temporal dynamics directly, or indirectly via environmental filters that drive local compositional patterns that are functionally distinct subsets of the regional species pool. In b) a framework (modified from [26]) for quantifying different types of temporal variation shows how change can be either directional or stable, and stable changes can occur either via high compositional change in composition, or via large temporal fluctuations in the amount of compositional dissimilarity. Directional change is quantified using the regression slope of the relationship between compositional dissimilarity and the square root of the time lag (or interval between sampling points) and compositional change is quantified using the average dissimilarity values for a plot. Temporal fluctuation is quantified using the root mean square of the residuals from the linear regression fit between dissimilarity and time lag. Temporal fluctuation reflects the amount of change in composition that occurs irrespective of directional change. This metric summarizes the variability in dissimilarity, and can have a large value even when the slope of the regression relationship is zero. Small values indicate high predictability in temporal variation which could occur either due to predictable directional change or constant compositional change where the amount of community change is similar regardless of time lag between sampling points. Large values indicate large fluctuations that are not well predicted by the time interval between sampling points.

Three primary mechanisms could generate spatial variability in temporal dynamics. First, spatial variation in external drivers may influence temporal dynamics directly at the landscape scale (Fig 1a). Second, local abiotic factors can interact with external drivers to either buffer or magnify their effects, producing spatial variability in how plant communities experience similar climate or disturbance. Finally, abiotic factors can act as strong environmental filters that limit the pool of species that is likely to occur [19,20], and variation in plant responses to climate and disturbance can alter how communities shift through time [21,22]. For example, recent evidence suggests that plant communities on infertile soils may be relatively resistant to climate variation because these communities contain ‘stress tolerant’ functional traits that constrain their ability to respond to climate [22,23]. Differences in functional composition across sites are more likely to reflect environmental filtering than other community assembly mechanisms because they are caused by differences in the presence or abundance of multiple species. For example, dispersal and demographic stochasticity [19,20,24] can alter the presence or abundance of a single species at a site, but are unlikely to affect suites of species in the same way.

Depending on conditions, ecological communities can either remain stable or shift in response to disturbance or a changing climate, causing directional change in composition [8,25,26]. In the absence of directional change, stability can occur either because the amount of compositional change (or appearance and disappearance of species) from year-to-year is highly predictable [27], or when a community exhibits a ‘loose equilibrium’ in which structure is stable over the long term despite year-to-year fluctuations in composition and abundance [8,25,26]. Temporal compositional change is likely to be influenced by local abiotic conditions such as resource supply, which can create sources and sinks on the landscape that vary in rates of local appearance and disappearance in species [15]. For example, there is some evidence that temporal turnover in composition may increase with productivity [28]. In contrast, temporal fluctuation may reflect species sorting mechanisms. For example, sites dominated by species with strong storage ability (such as perennial life forms, or seed banking forbs) can persist through unfavorable years [5,6,7], which could limit fluctuation from year to year. Alternatively, compensatory dynamics may increase temporal fluctuations at sites dominated by a few competitively superior annual species that respond strongly to interannual variations in precipitation [29,30]. Thus, teasing apart fluctuation-based temporal variation from high rates of compositional changemay reveal underlying mechanisms that operate differently depending on site-specific intrinsic factors.

Here, we evaluate the effects of external drivers and intrinsic factors on temporal dynamics using an 11-year dataset of understory vegetation composition from an oak-dominated landscape in California’s Sierra Nevada foothills. Grasslands and the intermingled savannas are dominated by exotic annual grass and herbaceous species, mostly from the Mediterranean [31]. This predominately annual ecosystem responds quickly to changes in disturbance and precipitation, making itideal for evaluating temporal dynamics. Water availability is a primary limiting factor and interannual fluctuations in species composition are linked to large variations in precipitation across years [32,33,34]. Additionally, fire and grazing are important sources of disturbance [35]. Grasslands on fertile soils are dominated by a small number of exotic annual grasses that suppress annual forb abundances by forming a dense layer of recalcitrant litter that inhibits forb germination [32,34]. Many of the non-native annual grasses do not create a persistent seed bank [36], and large interannual fluctuations in composition of these species have been observed in other studies [34,37]. Native perennial grasses and annual forbs persist in low abundance, and these species do better on soils with low fertility [38].

We compare grasslands where disturbance was experimentally manipulated using a prescribed burn and grazing removal. The study included a pre-treatment period where all plots were monitored for four years at moderate levels of livestock grazing. We use abiotic data, pre-treatment functional composition, and post-disturbance trajectories to tease apart the relative influence of disturbance, environmental filtering, and abiotic conditions on temporal dynamics. We predicted that: 1) external drivers (disturbance) affect temporal dynamics, causing more directional change at sites where disturbance regimes were altered during the course of the study compared to where disturbance remained constant, 2) local abiotic conditions and functional composition (reflecting environmental filtering) influence the magnitude of temporal fluctuation and compositional change at a plot. In particular, we expected that local abiotic conditions and functional composition should cause spatial variability in temporal dynamics even where external drivers were similar, creating hotspots on the landscape that are particularly responsive.

## Methods

### Ethics statement

A field permit for this work was provided by the Sierra Foothills Research and Extension Center, a facility owned and operated by the University of California. All plots for this study were located on the property of the extension center. No protected species were sampled during this study.

### Study site

This study was conducted in the northern Sierra Nevada foothills at the University of California Sierra Foothill Research and Extension Center. The center is located approximately 30 km east of Marysville, Yuba County, California (39° 15’ N, 121° 17’ W). The precipitation is Mediterranean with cool moist winters and hot dry summers. Mean annual precipitation is 730 mm and temperature is 15°C, and elevation ranges from 210 to 580 meters. Tree cover is dominated by blue oaks (*Quercus douglasii*) mixed with interior live oak (*Q*. *wislizeni*) and foothill pine (*Pinus sabiniana*). The patchy distribution of trees creates a mosaic of open grassland, savanna and woodlands [39]. Exotic annual grasses are dominant in both the understory and adjacent grasslands.

### Disturbance

The study area was divided into three adjacent watersheds (32–120 hectares), all grazed by cattle for decades before the study began. A total of 54 permanent plots were established in 1998 across the three watersheds using a stratified random design. All watersheds were grazed at a moderate level leaving an average of 157 kg per hectare per year of fall residual dry matter for the first four years of the experiment. In 2001, additional fencing was installed to exclude livestock from one watershed (including 21 plots) for the remainder of the study. The remaining two watersheds (including 16 and 17 plots) were grazed annually each winter and spring for the duration of the study. One of the annually grazed watersheds (16 plots) also received a prescribed burn in November of 2004. The fire was relatively low intensity, and incomplete in some plots. Using results of an ocular survey conducted after the burn, we re-categorized five plots that were less than 70% burned as grazed without a burn treatment. The experimental treatments included three categories, and plots were either grazed, fenced to exclude grazing, or burned and grazed.

The logistical challenges associated with manipulating grazing and fire across entire watersheds made it unfeasible to include multiple replicates of each disturbance type. Therefore, plots within a watershed were spatially closer to each other, introducing pseudo-replication into the study design [40]. The assignment of treatments to the three watersheds was selected randomly, and was unrelated to vegetation composition or abiotic variables. However, temporal dynamics could be incorrectly linked to treatments if underlying gradients in other factors (abiotic or biotic) that drive temporal dynamics varied among watersheds. Therefore, we examined whether the watersheds were inherently different from each other in the abiotic conditions or pre-treatment vegetation composition using Analysis of Variance and χ^2^ tests (full results are presented in S2 and S3 Tables, S1, S2 and S3 Figs). We found that the three watersheds were similar to each other in tree cover, most soil properties and pre-treatment species composition, with similar numbers of north and south facing slopes (S2 and S3 Tables, S1, S2 and S3 Figs). Watersheds are located on a hillside, with the fenced watershed at the lowest elevation, the grazed watershed at intermediate elevation, and the burned and grazed watershed at the highest elevation (r2 = 0.563, F-ratio = 35.2, P<0.001, S2 Table, see maps in S1 Fig). In addition, total Nitrogen roughly followed the elevational gradient, with high total N in the burned and grazed watershed, intermediate in the grazed watershed, and low in the fenced watershed (r2 = 0.153, F-ratio = 5.768, P = 0.005, S1 Table, S1 and S2 Figs).

To address the challenge of pseudo-replication in our modeling approach, we included watershed, total Nitrogen and elevation as random effects in all mixed modeling analyses. This allows relationships between explanatory and temporal variables to vary among watersheds and with elevation and total Nitrogen, accounting for the non-independence of data from spatially aggregated plots [41,42]. We cannot entirely eliminate the possibility that variables correlated with elevational or nitrogen gradients explain some of the variation in temporal dynamics. However, we have adopted as conservative an approach as possible to our analysis, and are confident that the effects of treatment on temporal dynamics cannot be entirely explained by elevation, underlying N gradients, or aggregation of plots in space.

### Sampling vegetation and abiotic variables

Vegetation was sampled within 54 plots along 10 meter transects each spring for 11 years between May 1^st^ and the beginning of July, near the peak of annual productivity. Each plot contained a single transect, and plots were permanently marked and revisited each spring. Plots were randomly located across the watershed within three categories of canopy cover (0–30, 30–60, >60%), defined using aerial photointerpretation using a 5 acre minimum mapping unit size (for spatial arrangement of plots, see S1 Fig). Along each transect the first plant species to be hit by lowering a point towards the ground was recorded every 10 cm [43], for a total of 100 points per plot. Non-vegetation data were removed from the dataset, and each species was relativized using the total number of vegetation points.

Environmental variables were recorded at each plot including aspect (north or south facing), slope, elevation, and topographic position (upper, middle and lower slope). Soils were collected at a depth of 10 cm in 2006 and analyzed for total Carlo-Erba Nitrogen, Bray Phosphorus [44], clay (%), and C:N ratio. Samples were analyzed at the UC Davis analytical lab (http://www.anlab.ucdavis.edu) with the exception of soil pH, which was measured at the UC Berkeley Range Ecology Laboratory.

### Quantifying temporal dynamics

To compare patterns of temporal variation in species composition, we used time lag analysis [26]. This approach is conceptually similar to methods used to quantify the distance decay in similarity [45], with the exception that plots are compared to themselves across years instead of to each other across space. We calculated dissimilarity values for each plot using Bray-Curtis dissimilarity. This metric varies from zero (total similarity) to one (total dissimilarity) and compares composition using differences in both relative abundance and species identity [46]. We chose this metric because it is appropriate for detecting underlying ecological gradients [47] and because it provides intermediate values when balancing the contribution of rare and abundant species [48]. Both species density and the relative abundance of species are known to affect biodiversity metrics [49], and the disadvantage of this metric is that it cannot distinguish between changes in abundance and the disappearance or re-appearance of a species. Additionally, because transect sampling of plants tends to over-represent the abundant species, we cannot tell whether the absence of a species from a plot is due to a true disappearance, or a plant that did not appear on the transect despite being present at low abundance. Therefore, all temporal dynamic variables incorporate the combined effects of changes in abundance and appearance/disappearance of species.

We used three methods to summarize trends in temporal dynamics (Fig 1b). Our goals were to distinguish between directional and non-directional change, and to quantify the magnitude of temporal fluctuation in a way that minimized the influence of directional change. Using linear regression, we related dissimilarity values to the square root of time lag which reduces the probability that smaller number of points at larger time lags will bias the analysis [26]. Dissimilarity and time lags included comparisons of each plot to itself over all possible combinations of years, yielding a total of 55 comparisons per plot. With this approach, a positive-sloped relationship indicates directional change [26] whereas a slope of zero indicates no directional shifts in composition (Fig 1b). We calculated the mean dissimilarity (indicating compositional change), the slope of the linear relationship between dissimilarity and time lag (indicating directional change), and the root mean square of the residuals from the linear regression line (indicating temporal fluctuation). The root mean square of the residuals quantifies the amount of fluctuation through time irrespective of directional change. This metric summarizes the variability in dissimilarity, and can have a large value even when the slope of the regression relationship is zero. Small values indicate high predictability in temporal variation which could occur either due to predictable directional change or constant compositional change where the amount of community change is similar regardless of time lag between sampling points. Large values indicate large fluctuations that are not well predicted by the time interval between sampling points. We did not expect compositional change and fluctuation to be correlated; a plot that predictably experiences 80% compositional change every year would yield a low value for temporal fluctuation. Alternatively, with large temporal fluctuations, dissimilarity will vary because some pairs of years will be more similar in composition whereas others will be very different, though average compositional change could be low.

### Landscape and local drivers

To determine whether patterns of temporal variability were related to landscape and local drivers, we related temporal dynamics variables to external drivers, abiotic factors and functional composition. We considered our predictions to be supported if 1) directional change was higher in plots where the disturbance regime changed during the study, and 2) if temporal fluctuation and compositional change was best explained by a combination of abiotic and functional composition. In particular, we expected that annual grasses would be associated with high temporal fluctuation, whereas high perennial and forb composition would be associated with low fluctuation.

**Fig 2:**
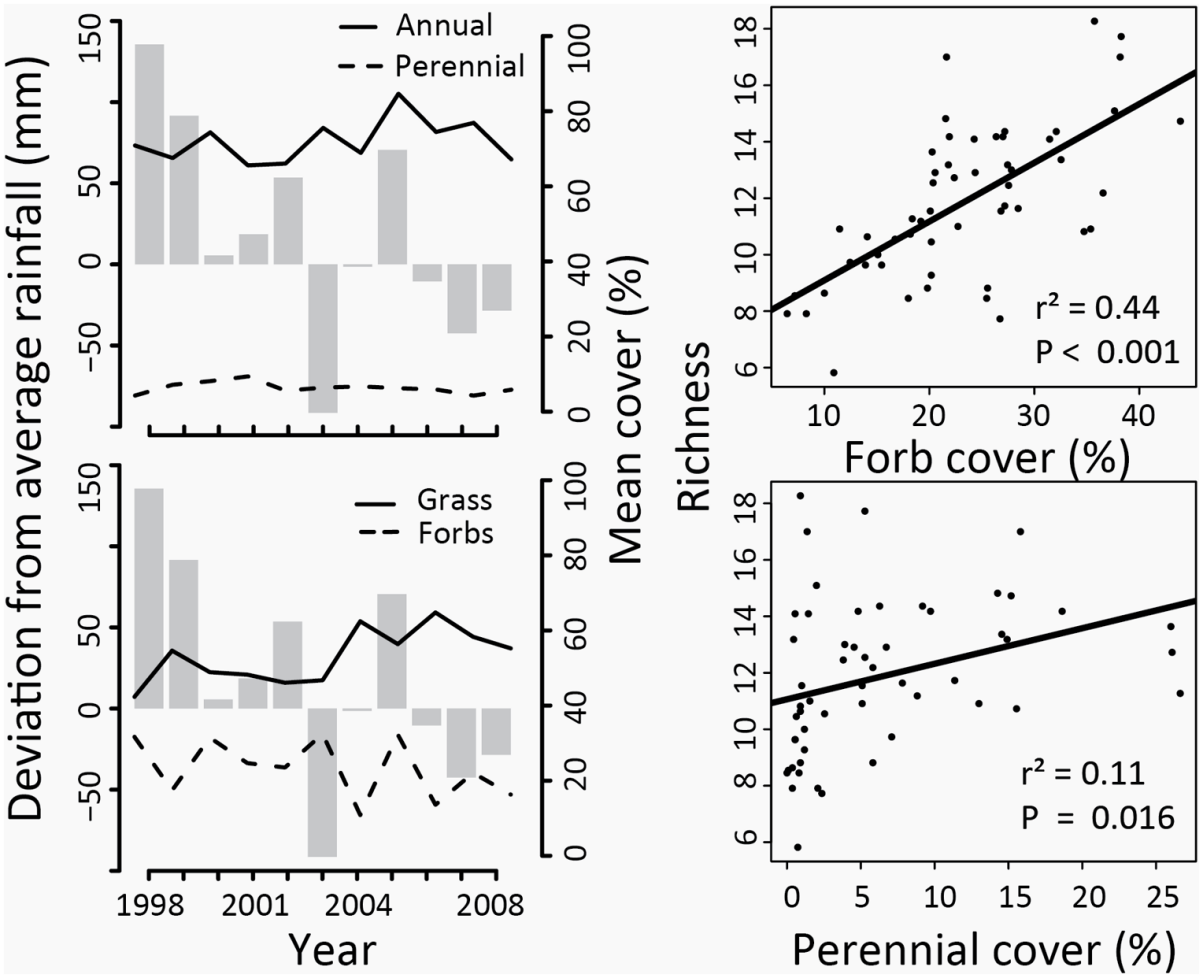
Functional composition, precipitation, and species richness. In the left column, changes in average grass, forb, perennial and annual composition across all study plots during the study period (in average percent cover) are represented as lines and bars represent deviations from the 20 year average in fall rainfall (mm) for each year during the study period. The right column shows the relationship between average perennials and forb composition and species richness. Points represent average cover or richness during the entire study period from 54 plots.

External driver variables were represented by treatment type (continuous grazing, grazing removal and burning). Abiotic factors included soil properties (clay (%), phosphorus, pH, C:N ratio) and environmental variables (aspect, topographic slope and position). Biotic variables that could reflect environmental filtering that limits the species pool at a site were represented using functional categories including grass, forb, perennial and annual composition. While these groupings represent only coarse functional categories, they are nonetheless highly relevant to temporal dynamics in California grasslands, where annual composition can fluctuate widely with interannual variation in precipitation [34], perennial composition tends to be more stable [23], and forbs are usually found at very low abundance, but often represent a large proportion of the species richness (Fig 2). Compositional variables were quantified using the species composition of each plot before disturbance treatments were applied, averaged over the pre-treatment period. Because functional composition variables were correlated with one another (especially grass and annual composition), we included only pre-treatment forb and perennial composition, which were not correlated. These two variables are highly negatively correlated with the absent variables and with species richness; high forb composition also indicates high species richness and low grass composition, and high perennial composition indicates relatively low annual composition (Fig 2).

### Statistical approach

We used linear mixed modeling to identify pathways associated with temporal dynamics. All models were fit with gamma diversity nested within total Nitrogen, elevation, and watershed as random effects, and directional change, temporal fluctuation or compositional change as response variables. Gamma diversity was represented by the total number of species that occurred at each plot over 11 years, and was included as a random effect because beta diversity metrics (including Bray-Curtis) are known to be related both alpha and gamma diversity [50]. This is problematic because forbs account for the majority of the species richness in this system (Fig 2). As a result, the pre-treatment forb composition is strongly related to total plot richness, which is in turn related to Bray-Curtis dissimilarity metrics. We also evaluated the potential for multi-colinearity in intrinsic factors using Pearson correlations with a cutoff of r > 0.5. There were only a few cases of problematic correlations (r > 0.5 for tree cover and perennial cover and for Carbon and Nitogen), and where they occurred, we eliminated one of the two correlated variables (tree cover and total Carbon) from the analysis.

We used backwards stepwise selection using AIC and guidelines provided in [41] to obtain a final best fitting model for each temporal dynamic variable (S1 Table). To evaluate the effects of disturbance and aspect on temporal variation, we used Tukey post hoc multiple comparison tests with p-values adjusted using the Westfall method for correction. Adjusted p-values are significant at the level of p = 0.05 [51]. Models were validated using graphical inspection of residuals. All analyses were performed using R 3.1.1 [52] with the package nlme [53]. Distance matrices were computed using the package vegan [54], and multiple comparison post hoc tests were computed using the glht function in the multcomp package [55]. All data used for this analysis is included as a supplement to this paper (S1 Database).

## Results

A total of 162 species were identified during the study period, with an average of 12.1 species identified at each plot per year. The most abundant species were annual grasses, including *Cynosurus echinatus*, *Bromus madritensis* and *Festuca perennis*. Common forbs included *Trifolium hirtum*, *Torilis nodosa*, and *Carduus pycnocephalus*, and common perennials included *Elymus glaucus*, *Bromus carinatus*, and *Galium bolanderi*. The species identified in the study period were 72.2% forbs, 20.9% grasses, and 5.5% shrubs. Annuals formed the majority of taxa, 53.7% of all species; the rest were 34.6% perennial, 9.25% unknown and 1.8% facultatively annual or perennial. Forbs accounted for most of the diversity, but grasses dominated the cover in all years (Fig 2). During the study period, total rainfall varied between 384 and 1,517 mm (Fig 2), fall rainfall from 17 to 244 mm, winter rainfall from 115 to 612 mm, and spring rainfall from 21 to 308 mm.

**Fig 3:**
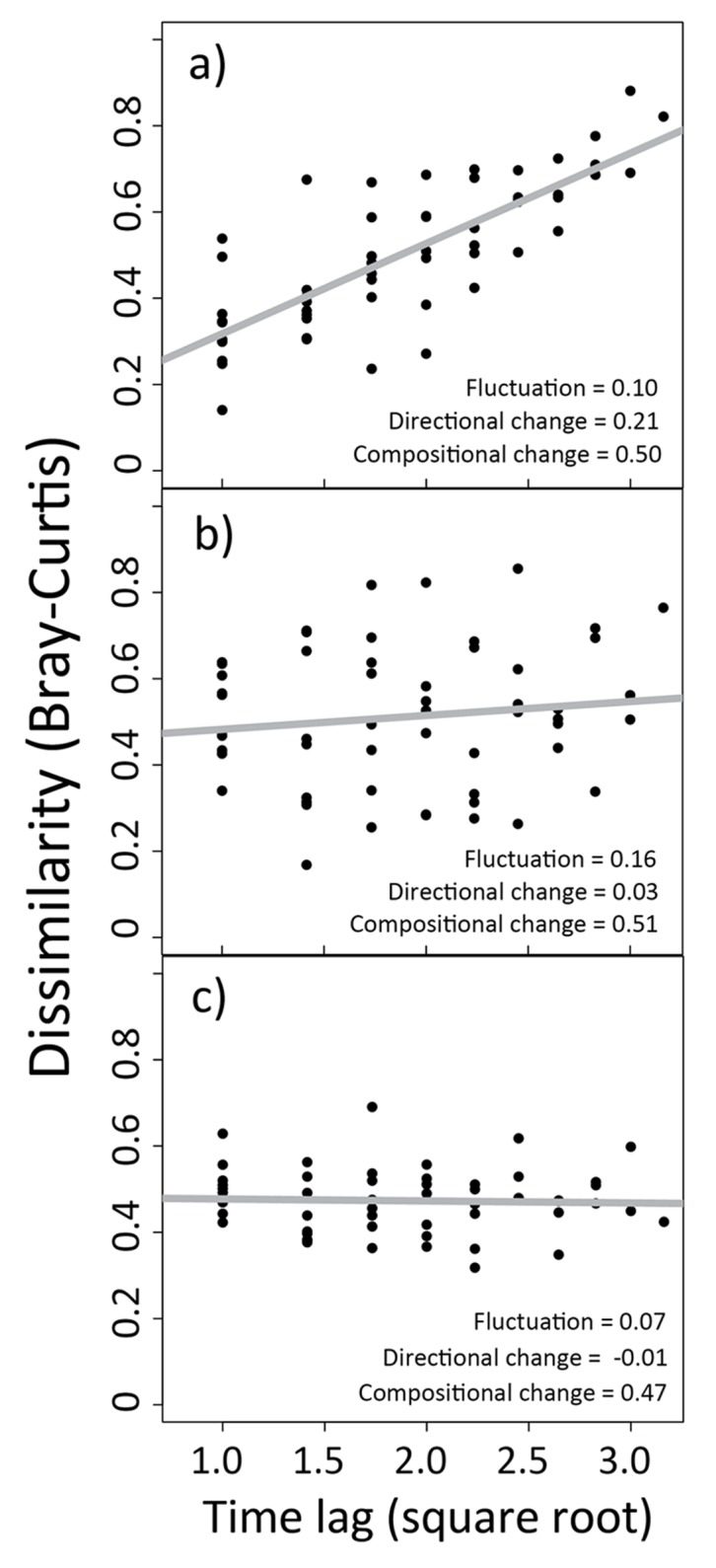
Variation in temporal dynamics. Temporal dynamics on three plots representing different patterns in temporal dynamics. Axes show the relationship between the square root of time lag (number of years between sample points) and Bray-Curtis dissimilarity in species composition. For visual clarity, only three example plots are shown. Patterns show a) directional change, b) high temporal fluctuation without directional change, and c) high compositional change with low temporal fluctuation and without directional change. See Fig 1b for an explanation of each type of temporal pattern.

Temporal dynamics varied considerably from plot to plot (Fig 3). Of 54 plots, 35 showed evidence of significant directional change (quantified as a P value < 0.05 for the regression slope between time and dissimilarity). Regression slopes varied between -0.00068 and 0.21. Temporal fluctuation, quantified as root mean square residual values, varied from a minimum of 0.07 on plots with the least fluctuation to 0.189 on plots with maximum fluctuation. Compositional change, quantified as mean Bray-Curtis dissimilarity, varied between 0.29 and 0.56, corresponding to an average compositional change of between 29 and 56 percent. Directional change, temporal fluctuation and compositional change were uncorrelated with one another.

### Landscape and local drivers

**Fig 4:**
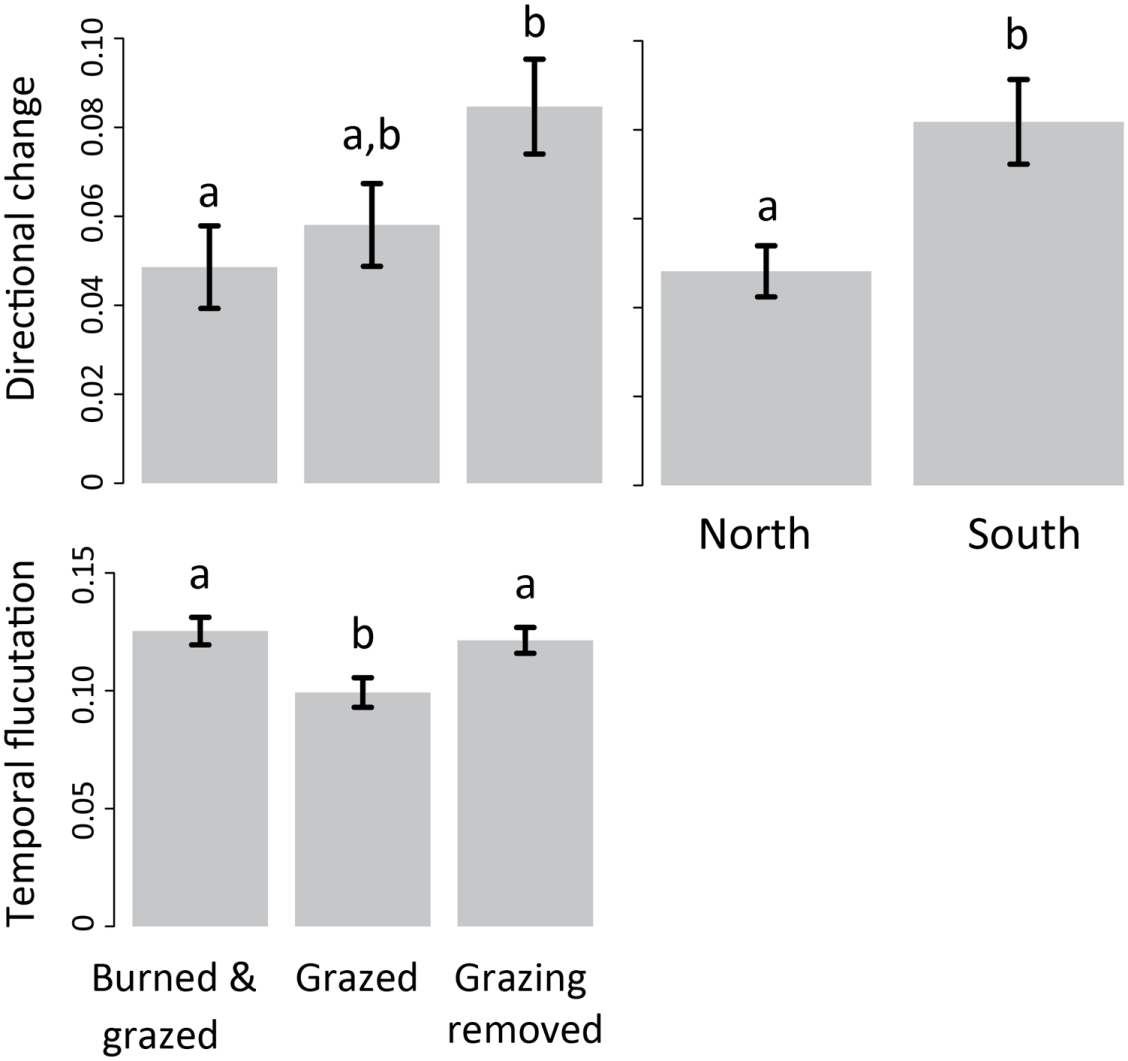
Disturbance and aspect. The effects of disturbance (grazing removal, burning) on directional change and temporal fluctuation, and the relationship between aspect (north or south facing) and directional change where bars represent means (± 1SE). Directional change is the slope of the relationship between the square root of time lag (or time between sampling points) and Bray-Curtis dissimilarity in species composition for each plot. Temporal fluctuation is quantified as the root mean square of the residuals from the linear regression line relating time lag to dissimilarity in species composition for each plotSignificant relationships are indicated with different letters.

**Fig 5:**
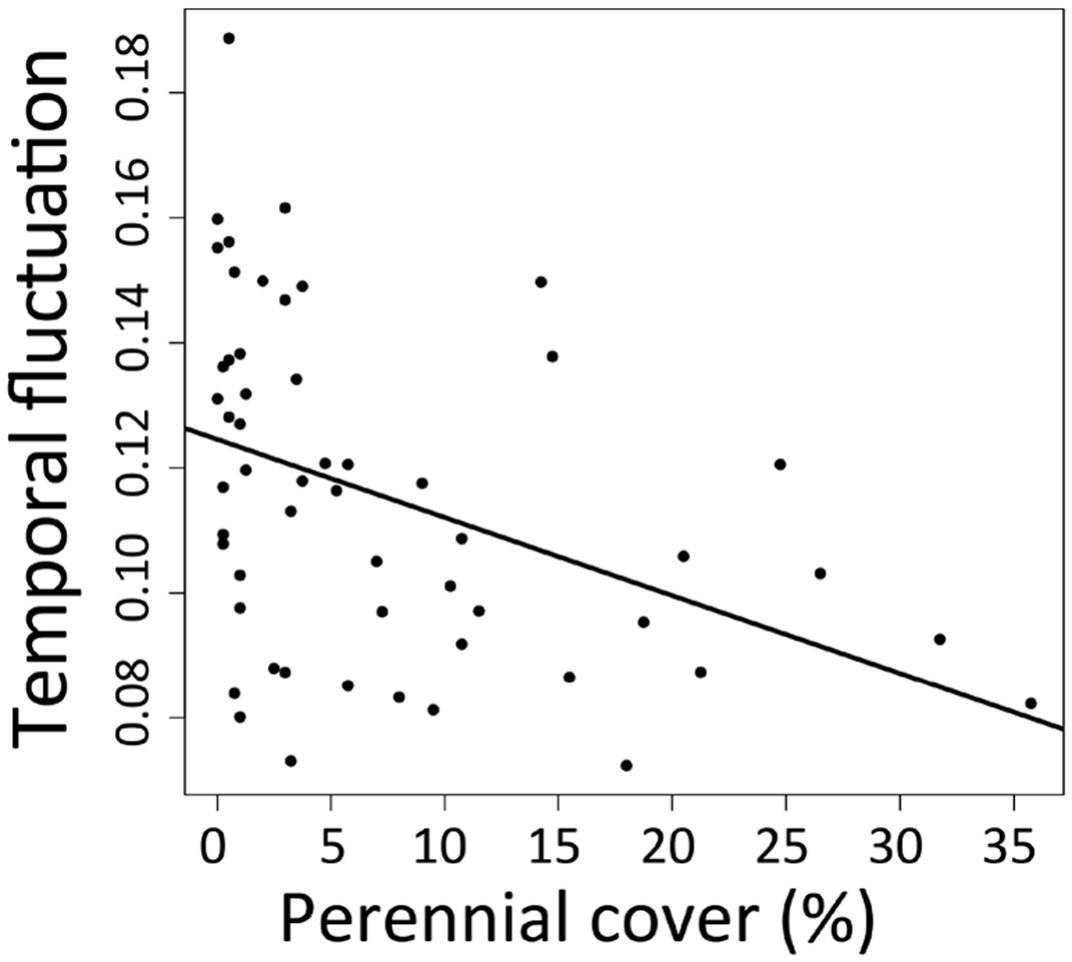
Temporal fluctuation and functional composition. Relationships between temporal fluctuation and functional composition (perennial cover %)during the pre-treatment period between 1998 and 2001 when a fence was installed to exclude grazing from one experimental watershed. Temporal fluctuation is quantified as the root mean square of the residuals from the linear regression line relating time lag to Bray-Curtis dissimilarity in species composition, calculated at the plot level.

**Fig 6:**
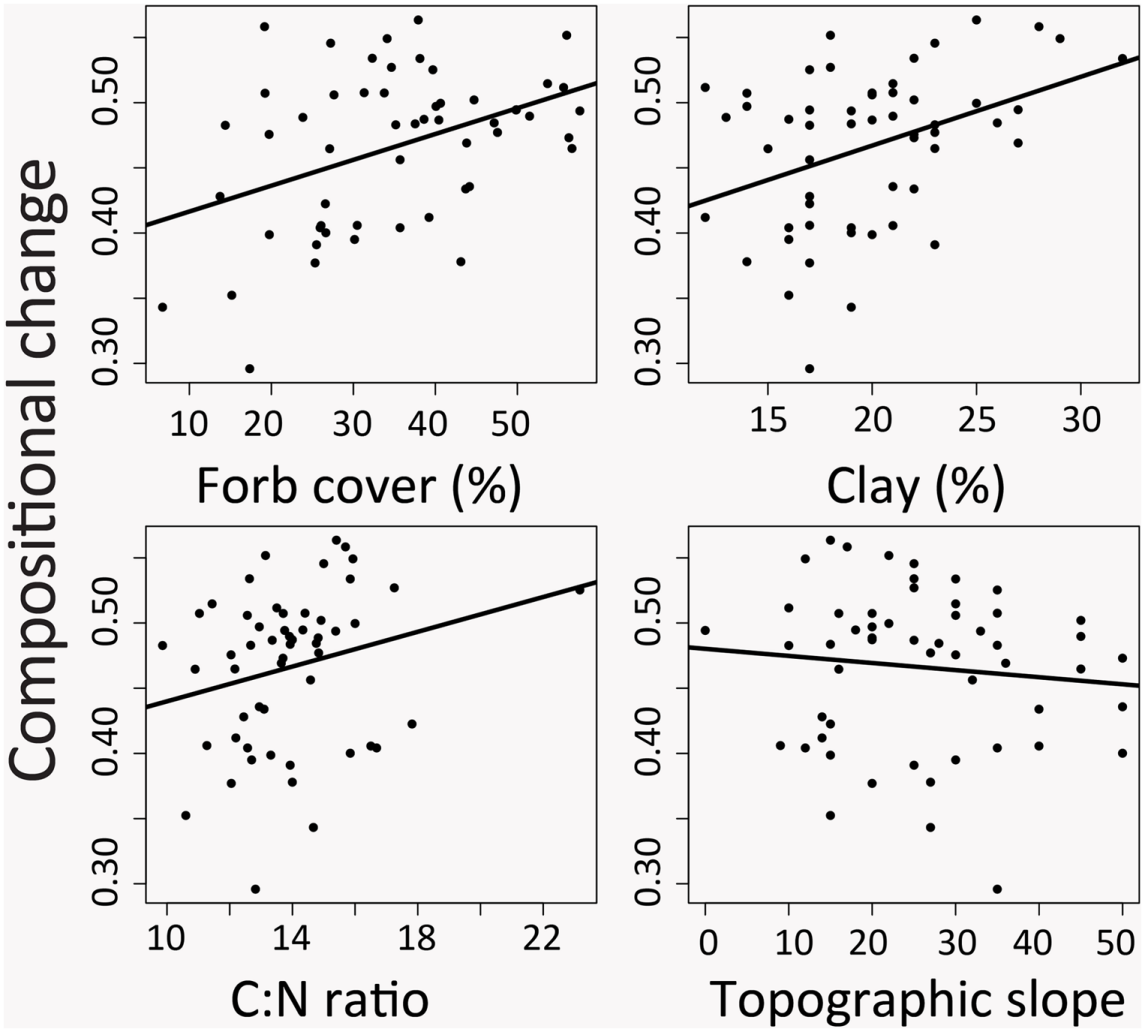
Compositional change, abiotic factors and functional composition. Relationships between compositional change, abiotic factors (soil clay, soil C:N ratio, topographic position) and functional composition (forb cover %) during the pre-treatment period between 1998 and 2001 when a fence was installed to exclude grazing from one experimental watershed. Compositional change is the average Bray-Curtis dissimilarity in species composition, calculated across all years in the study (1998–2008) for each plot.

Both directional change and temporal fluctuations were related to disturbance type (Fig 4). Directional changes were most pronounced on plots where grazing was removed, and fluctuations were least pronounced on plots where disturbance regime remained constant during the study period (continuous grazing). All three temporal variables were best predicted using models that included local abiotic variables, demonstrating strong spatial variation in temporal dynamics. Temporal fluctuation and compositional change depended on functional composition in the pre-treatment period (Figs 5 and 6), indicating that functional composition plays a role in how communities respond to external drivers. Directional change and temporal fluctuations were best predicted using a combination of external drivers (disturbance) and local variables (Figs 4 and 5), while compositional change was influenced only by local abiotic variables and functional composition (Table 1, Fig 6). Directional change was related to local abiotic factors but not functional composition, and was most pronounced on south-facing slopes (Table 1, Fig 4). Compositional change was unrelated to disturbance, and was highest on plots with high clay, C:N ratio, and forb composition as well as low topographic slope (Fig 6). Temporal fluctuation was greatest at plots with low perennial cover, and where disturbance was modified during the study period, either with burning or grazing removal (Figs 4 and 5).

**Table 1:** Predictors of temporal dynamics. Predictors of three types of temporal dynamics (temporal fluctuation, directional change, and average compositional change), including disturbance, local abiotic factors, and functional composition. Estimates are taken from best fitting linear mixed models.

	Estimate	SE	DF	*t*	*P*	
***Directional change (regression slope)***						
Intercept	0.070	0.027	27	2.612	0.015	*
Grazing	0.014	0.014	27	1.028	0.313	
Grazing removal	0.032	0.013	27	2.436	0.022	*
Southern aspect	0.033	0.011	27	2.930	0.0068	*
Clay (%)	-0.002	0.001	27	-1.523	0.139	
***Temporal fluctuation (RMS residuals)***						
Intercept	0.148	0.010	27	14.36	<0.001	*
Grazing	-0.025	0.0080	27	-3.06	0.0049	
Grazing removal	-0.007	0.0074	27	-1.00	0.33	
Forb	-0.038	0.026	27	-1.44	0.16	
Perennial	-0.0012	0.00036	27	-3.37	0.0023	*
***Compositional change (mean Bray-Curtis distance)***			DF	t-value	p-value	
Intercept	0.303	0.055	25	5.543946	<0.001	*
Southern aspect	0.021	0.015	25	1.392442	0.18	
Slope	-0.0014	0.00056	25	-2.57353	0.016	*
Topographic position	-0.012	0.0067	25	-1.73251	0.096	
Clay (%)	0.0032	0.0015	25	2.143758	0.042	*
C:N ratio	0.0068	0.0030	25	2.274379	0.032	*
Forb	0.169	0.057	25	2.963612	0.0066	*

Asterisks (*) indicate significant variables (P < 0.05) in each model.

## Discussion

We found evidence for spatial variability in temporal dynamics, reflecting the influence of multiple factors operating at landscape and local spatial scales. The combined effects of landscape-level external drivers, local abiotic factors and environmental filtering created site-specific dynamics that could only be predicted by quantifying multiple types of temporal dynamics. Response to disturbance was not uniform, but depended on local abiotic factors. Furthermore, abiotic factors alone did not predict temporal dynamics; instead, plots contained subsets of the total species pool that responded differently to external drivers.

Our results indicate that hotspots of community responsiveness to disturbance and changing climate will occur where the effects of external drivers are magnified by local abiotic conditions. For example, rapid community shifts in response to a change in disturbance occurred on south-facing slopes, where directional change was greatest. Likewise, fluctuating composition was particularly extreme on plots with high and high annual composition, while compositional change was higher on plots with high clay content and high C:N ratios. Water is a major limiting factor in semi-arid ecosystems [56]. In a recent study conducted in one of the same watersheds as our study [57], seasonal variation in soil water content depended on canopy cover, aspect, and the presence of a clay pan beneath the upper soil horizon. Reduced soil water holding capacity on soils low in clay and on south-facing slopes [58] may magnify wet-dry cycles [59], and could be responsible for the effects on temporal dynamics. Higher fluctuations and compositional change on plots with high C:N ratios and clay suggest that resources play a key role in mediating temporal dynamics. Greater resource availability improves plant performance, and may increase colonization rates via increased dispersal potential of plants in the local species pool [60]. We also found lower directional change on north-facing slopes, possibly indicating stronger resilience to disturbance on plots with greater moisture storage.

Changes to disturbance regimes, especially grazing abandonment, altered temporal dynamics, shifting patterns from stable compositional change and fluctuation to directional change. Livestock exclusion can change species composition in many types of grasslands, and is often associated with a decline in species richness [56,61,62], or directional change in composition [25,63]. Directional change was not affected by the prescribed burn, possibly because the burn was a low intensity fire conducted during a single year. The timing of the burn also makes directional change more difficult to detect in burned plots than in plots where grazing was removed because there are larger numbers of pairs of years in the pre-treatment period, when directional change is not expected to have occurred. Therefore, it is possible that some directional change did occur that we did not detect. However, in California, a reduction in the abundance of grasses in the months following a fire is common, but is rarely sustained beyond the first year [35]. Fire did increase temporal fluctuation in burned plots compared to plots with continuous grazing. This could have occurred via interactions with grazing [8]. Fire can attract livestock in the post-burn period, and because livestock grazing is asynchronous in space and time [8], selection of burned patches in one year could lead to compositional changes that do not persist unless livestock return to the same plot in subsequent years.

We found evidence that temporal dynamics are driven in part by variation in how different functional groups respond to external drivers, suggesting that environmental filtering shifts the local species pool at a plot, producing sets of species that do not change in the same way over time. Other community assembly drivers can also influence the species pool at a plot. For example, demographic stochasticity, priority effects, and dispersal barriers can all influence the subset of species present at a plot, irrespective of abiotic characteristics [19,20,64]. However, while these mechanisms are likely to influence the presence of a single species, it is unlikely that they would influence the functional composition of entire groups of species such as all forbs or all perennials, as in our study. Thus, we conclude that functional differences between plots during the pre-treatment period are much more likely to be driven by environmental filtering than by other community assembly mechanisms. At our study site, forbs have lower overall abundance, but account for much of the species richness whereas grasses are dominant, fluctuate in abundance at a plot from year to year, and account for less of the overall species richness. Other work in California grasslands has documented higher rates of colonization and extinction at species rich sites with high numbers of seed-banking forbs [65], as well as less spatial consistency from year to year with forbs than other functional groups [66]. Thus, greater forb composition may contribute to higher compositional change because forb-dominated sites contain a greater proportion of transient species. Temporal fluctuation was negatively related to perennial composition, probably because long life spans enable perennials to buffer the effects of climate variability [6]. Grasses are known to respond strongly to precipitation in California [34,67], and temporal fluctuations are likely a signature of response to this variability since plots with low perennial composition tended to be dominated by annual grasses. Many of the annual grasses in our system do not create strong seed banks [36] which could substantially reduce the buffering capacity at plots that are grass dominated. Dominance by a single life history type may also reduce colonization opportunities for other species, leading to higher fluctuation in response to variation in precipitation [21].

Both local and landscape variables predicted temporal dynamics, creating spatial variation in how plots changed through time. Other studies have also documented spatial variation in temporal dynamics. For example, in North American tallgrass prairie, Collins [25] found variation in the amount of directional change between sites. In a Mongolian grassland, He *et al*. [63] found that response to long-term grazing exclusion depended on the grassland community type, due to variation in the accumulation of litter. In California grasslands, Fernandez-Going *et al*. [23] found that stress-tolerant species associated with serpentine soils did not respond to climate as easily as species on non-serpentine. Finally, at the global scale, Korhonen *et al*. [12] found faster compositional change in the tropics and with larger organisms in aquatic systems. The main focus of most of this work has been on patterns at a broad geographic scale, or a single temporal driver such as compositional change, often without considering how factors at multiple spatial scales might affect fine scale dynamics. Our study takes a different approach; by simultaneously including processes at both the landscape and local scale, our results reveal how multiple mechanisms can create strong context-dependency in temporal dynamics. Furthermore, evaluating multiple types of temporal dynamics reveals that community shifts, stability, and fluctuations are not driven by the same sets of factors.

Spatial variability in temporal dynamics reflects the joint importance of environmental heterogeneity, environmental filtering, and disturbance. We show that the controls on temporal dynamics cannot be accurately understood without considering drivers at multiple spatial scales. Annual-dominated grasslands are well suited to evaluating temporal variability because responses to precipitation and disturbance are rapid. While it is likely that these results are applicable to other systems, establishing their generality will require additional studies, and longer time-series’ may also be necessary.

## Supporting Information

S1 Database Temporal dynamics and environmental data. Dataset used for analysis where directional change, turnover and fluctuation columns represent temporal dynamics variables, and all other columns represent environmental variables and pre-treatment richness and functional composition.
(XLSX)

S1 Fig Map of correlated environmental variables. Map of study area showing the boundaries of three watersheds, treatment applications and year of treatment, and 54 sampled plots. Diameter of plot circle indicates Elevation (m), total soil Nitrogen (ppm), and total soil Carbon (ppm).
(TIF)

S2 Fig Treatments and environmental variables. Relationships between elevation (m), total Nitrogen (ppm) and total Carbon (ppm) and watersheds where: (1) Grazing was removed in 2000, (2) Grazing occurred annually for the duration of the study, and (3) Grazed occurred annually and a prescribed burn was conducted in 2004. Explanatory variables are from 54 study plots distributed across the three watersheds. Values for r^2^, F ratios and P-values are from Analysis of Variance quantifying the relationship between each variable and the treatment type. Unfilled point show raw data, filled black point show averages, and error bars represent approximate confidence intervals (mean ± 2xSE).
(TIF)

S3 Fig Treatments and functional composition. Relationships between pre-treatment functional composition, as cover of annual, perennial, forb and grass and watersheds where: (1) Grazing was removed in 2000, (2) Grazing occurred annually for the duration of the study, and (3) Grazed occurred annually and a prescribed burn was conducted in 2004. Explanatory variables are from 54 study plots distributed across the three watersheds. Values for r^2^, F ratios and P-values are from Analysis of Variance quantifying the relationship between each variable and the treatment type. Unfilled point show raw data, filled black point show averages, and error bars represent approximate confidence intervals (mean ± 2xSE).
(TIF)

S1 Table Model selection results for three temporal dynamics variables. Model selection results from models of three temporal variables using linear mixed effects models with Gamma diversity, nitrogen, elevation and watershed as nested random effects. Model selection was conducted using backwards stepwise procedures on the basis of AIC. See methods section for more details on each variable.
(DOCX)

S2 Table Treatments and explanatory variables. Relationships between explanatory variables and treatment type (Burned and grazed, Grazed, or Grazing removed) applied to three watersheds. Explanatory variables are from 54 study plots distributed across the three watersheds. Values for r^2^, F ratios and P-values are from Analysis of Variance quantifying the relationship between each variable and the treatment type. Significant relationships are highlighted in bold.
(DOCX)

S3 Table Treatments and aspect and topographic position. Relationships between explanatory variables aspect (quantified as the deviation from a northerly aspect) and topographic position, and treatment type (Burned and grazed, Grazed, or Grazing removed) applied to three watersheds. Explanatory variables are from 54 study plots distributed across the three watersheds. Values for χ^2^ statistics, degrees of freedom, and P-values are taken from a χ^2^ test quantifying the relationship between each variable and the treatment type.
(DOCX)
