# Supplementary figures and images for: Hotspots of Community Change: Temporal Dynamics Are Spatially Variable in Understory Plant Composition of a California Oak Woodland
Source: PLoS One. 2015 Jul 29;10(7):e0133501. doi: 10.1371/journal.pone.0133501 (PMC4519272; doi:10.1371/journal.pone.0133501)

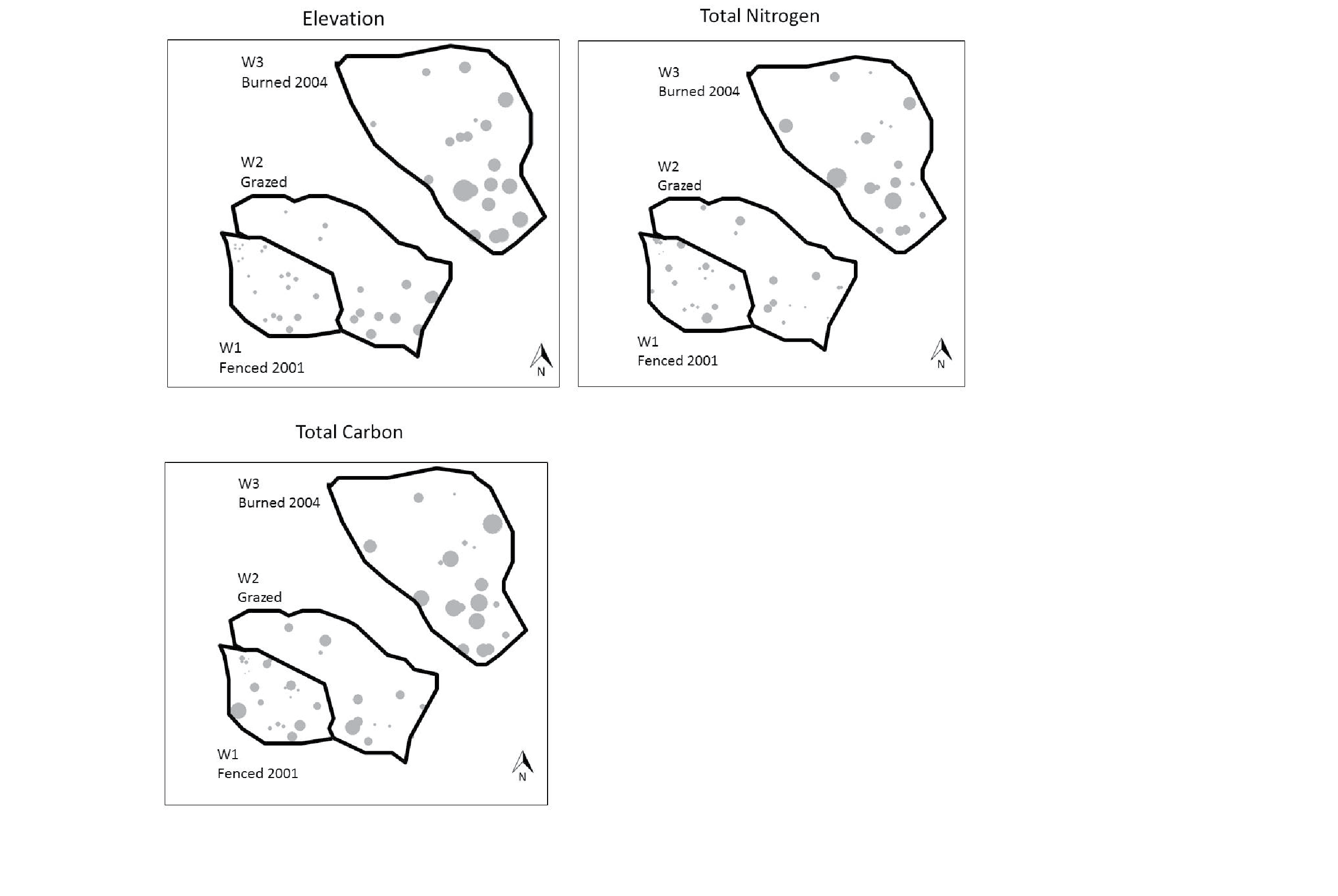

Supplement: S1 Fig — Map of study area showing the boundaries of three watersheds, treatment applications and year of treatment, and 54 sampled plots. Diameter of plot circle indicates Elevation (m), total soil Nitrogen (ppm), and total soil Carbon (ppm). (TIF) [file pone.0133501.s002.tif]

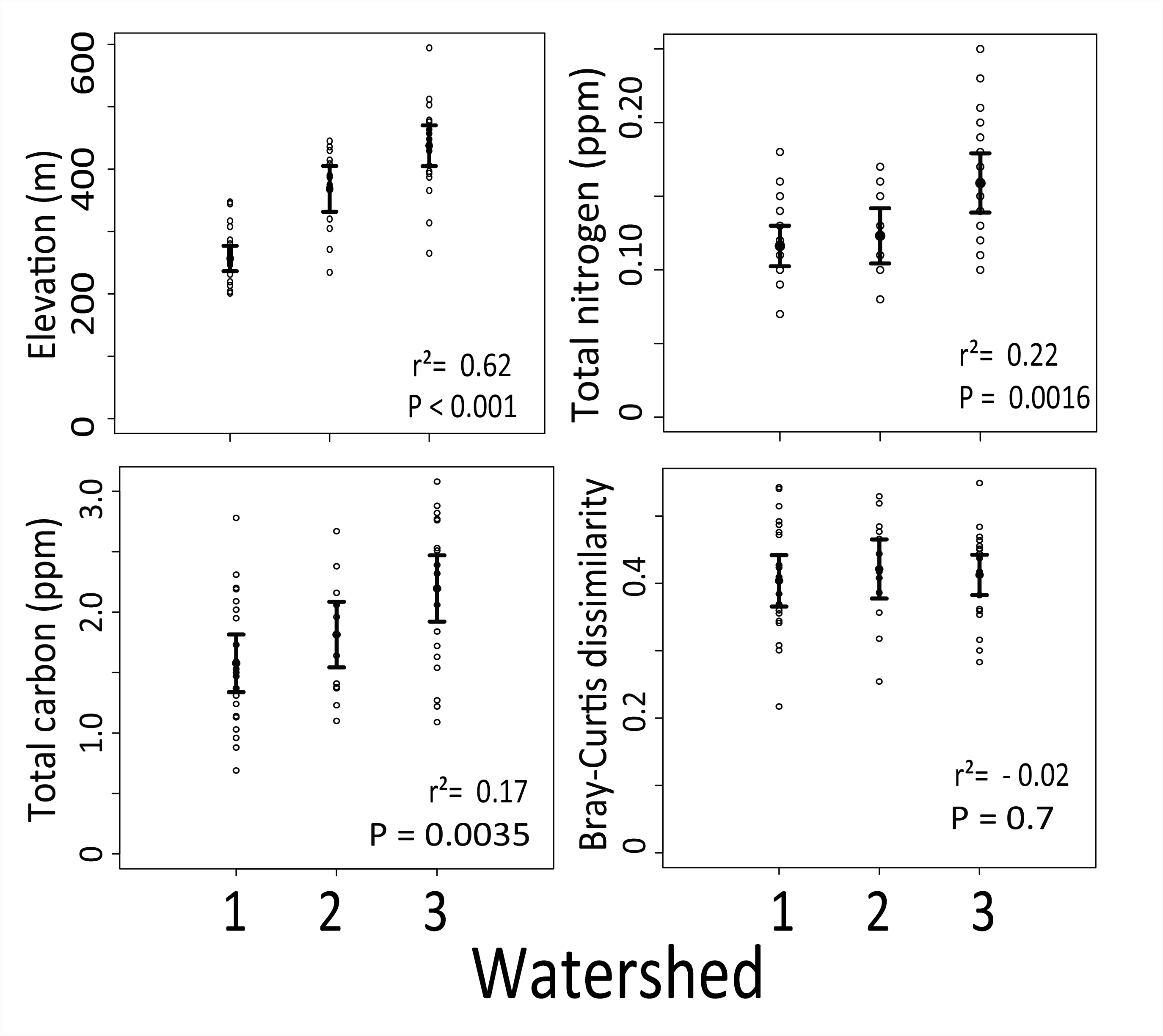

Supplement: S2 Fig — Relationships between elevation (m), total Nitrogen (ppm) and total Carbon (ppm) and watersheds where: (1) Grazing was removed in 2000, (2) Grazing occurred annually for the duration of the study, and (3) Grazed occurred annually and a prescribed burn was conducted in 2004. Explanatory variables are from 54 study plots distributed across the three watersheds. Values for r2, F ratios and P-values are from Analysis of Variance quantifying the relationship between each variable and the treatment type. Unfilled point show raw data, filled black point show averages, and error bars represent approximate confidence intervals (mean ± 2xSE). (TIF) [file pone.0133501.s003.tif]

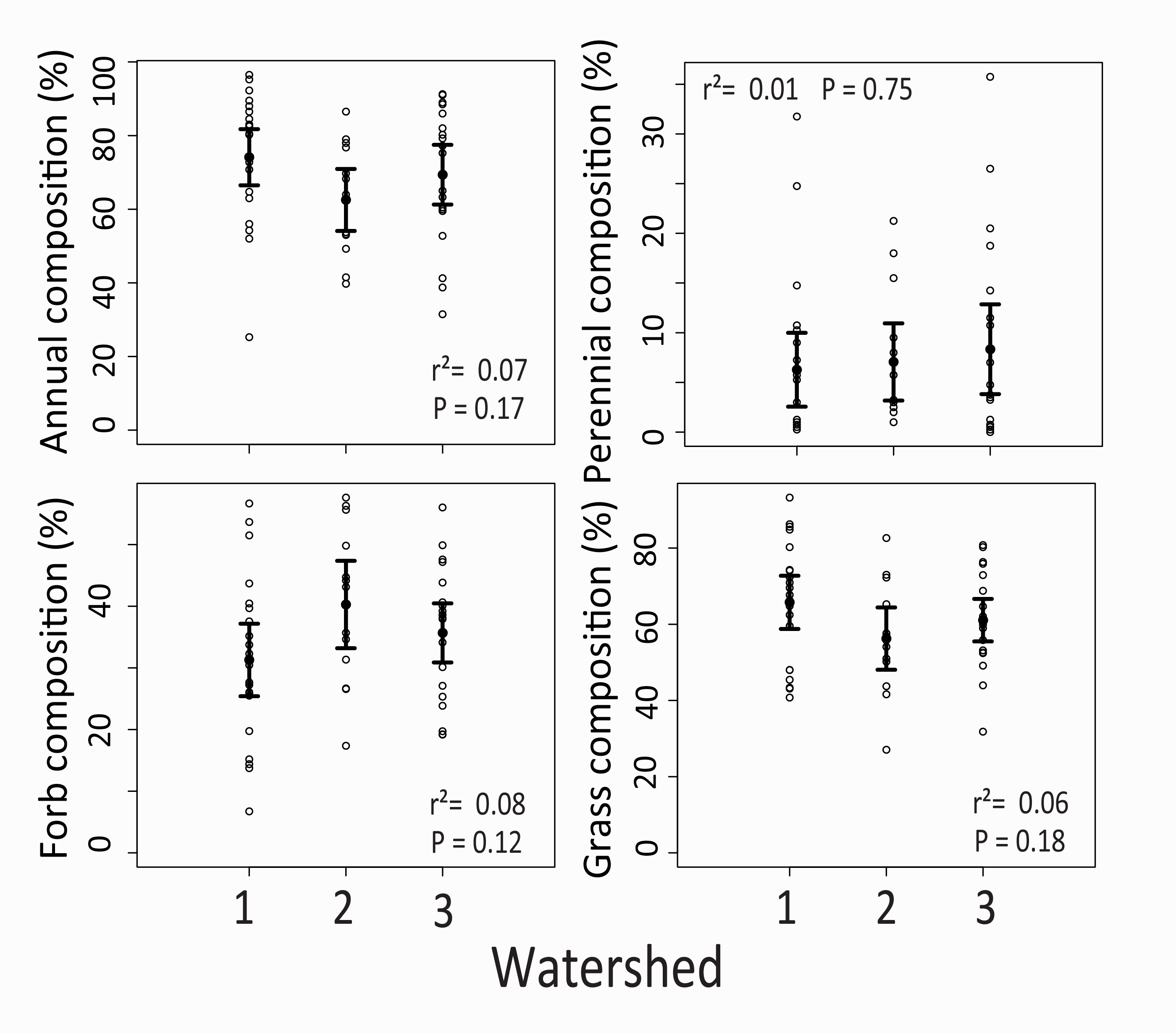

Supplement: S3 Fig — Relationships between pre-treatment functional composition, as cover of annual, perennial, forb and grass and watersheds where: (1) Grazing was removed in 2000, (2) Grazing occurred annually for the duration of the study, and (3) Grazed occurred annually and a prescribed burn was conducted in 2004. Explanatory variables are from 54 study plots distributed across the three watersheds. Values for r2, F ratios and P-values are from Analysis of Variance quantifying the relationship between each variable and the treatment type. Unfilled point show raw data, filled black point show averages, and error bars represent approximate confidence intervals (mean ± 2xSE). (TIF) [file pone.0133501.s004.tif]
