# Supplementary material for: Hotspots of Community Change: Temporal Dynamics Are Spatially Variable in Understory Plant Composition of a California Oak Woodland
Source: PLoS One. 2015 Jul 29;10(7):e0133501. doi: 10.1371/journal.pone.0133501 (PMC4519272; doi:10.1371/journal.pone.0133501)
Supplement: S1 Table — Model selection results from models of three temporal variables using linear mixed effects models with Gamma diversity, nitrogen, elevation and watershed as nested random effects. Model selection was conducted using backwards stepwise procedures on the basis of AIC. See methods section for more details on each variable. (DOCX) [file pone.0133501.s005.docx]

**S1 Table**.

|  | Model | AIC | ∆ AIC |
| --- | --- | --- | --- |
| ***Directional change (Regression slope)*** | |  |  |
|  | Treatment + Aspect + Clay | -180.51 | 0 |
|  | Treatment + Aspect + Clay + C:N ratio | -179.64 | 0.87 |
|  | Treatment + Aspect + Slope + Clay + C:N ratio | -178.28 | 2.23 |
|  | Treatment + Aspect + Slope + Clay + C:N ratio + Perennial | -176.68 | 3.83 |
|  | Treatment + Aspect + Position + Slope + Clay + C:N ratio + Perennial | -175.16 | 5.35 |
|  | Treatment + Aspect + Position + Slope + Clay + Phosphorus + C:N ratio + Perennial | -173.57 | 6.94 |
|  | Treatment + Aspect + Position + Slope + Clay + Phosphorus + C:N ratio + Forb + Perennial | -171.58 | 8.93 |
|  | Treatment + Aspect + Position + Slope + Clay + Phosphorus + pH + C:N ratio + Forb + Perennial | -169.58 | 10.934 |
| ***Temporal fluctuation (RMS residuals)*** | |  |  |
|  | Treatment + Forb + Perennial | -243.66 | 0 |
|  | Treatment + Phosphorus + Forb + Perennial | -243.06 | 0.6 |
|  | Treatment + Position + Phosphorus + Forb + Perennial | -242.07 | 1.59 |
|  | Treatment + Position + Phosphorus + C:N ratio + Forb + Perennial | -241.19 | 2.47 |
|  | Treatment + Position + Phosphorus + pH + C:N ratio + Forb + Perennial | -239.75 | 3.91 |
|  | Treatment + Position + Slope + Phosphorus + pH + C:N ratio + Forb + Perennial | -237.91 | 5.75 |
|  | Treatment + Aspect + Position + Slope + Phosphorus + pH + C:N ratio + Forb + Perennial | -235.92 | 7.74 |
|  | Treatment + Aspect + Position + Slope + Clay + Phosphorus + pH + C:N ratio + Forb + Perennial | -233.92 | 9.74 |
| ***Turnover (mean Bray-Curtis distance)*** | |  |  |
|  | Aspect + Position + Slope + Clay + C:N ratio + Forb | -163.79 | 0 |
|  | Aspect + Position + Slope + Clay + Phosphorus + C:N ratio + Forb | -162.7 | 1.09 |
|  | Aspect + Position + Slope + Clay + Phosphorus + pH + C:N ratio + Forb | -160.92 | 2.87 |
|  | Aspect + Position + Slope + Clay + Phosphorus + pH + C:N ratio + Forb + Perennial | -158.92 | 4.87 |
|  | Treatment + Aspect + Position + Slope + Clay + Phosphorus + pH + C:N ratio + Forb + Perennial | -156.04 | 7.75 |
