# Supplementary material for: Hotspots of Community Change: Temporal Dynamics Are Spatially Variable in Understory Plant Composition of a California Oak Woodland
Source: PLoS One. 2015 Jul 29;10(7):e0133501. doi: 10.1371/journal.pone.0133501 (PMC4519272; doi:10.1371/journal.pone.0133501)
Supplement: S2 Table — Relationships between explanatory variables and treatment type (Burned and grazed, Grazed, or Grazing removed) applied to three watersheds. Explanatory variables are from 54 study plots distributed across the three watersheds. Values for r2, F ratios and P-values are from Analysis of Variance quantifying the relationship between each variable and the treatment type. Significant relationships are highlighted in bold. (DOCX) [file pone.0133501.s006.docx]

**S2 Table**.

| Variable | r^2^ | F | P-value |
| --- | --- | --- | --- |
| Total nitrogen | 0.153 | 5.768 | **0.0055** |
| Total carbon | 0.136 | 5.185 | **0.0089** |
| Elevation | 0.563 | 35.200 | **<0.001** |
| Tree cover (%) | 0.018 | 1.493 | 0.234 |
| Topographic slope | 0.041 | 2.129 | 0.129 |
| Phosphorus | 0.007 | 1.179 | 0.316 |
| Clay (%) | -0.038 | 0.025 | 0.976 |
| Sand (%) | -0.275 | 0.291 | 0.749 |
| Silt (%) | -0.009 | 0.765 | 0.471 |
| C:N ratio | 0.028 | 1.750 | 0.184 |
| pH | -0.018 | 0.513 | 0.597 |
| Calcium | -0.037 | 0.040 | 0.961 |
| Forb cover (%) | 0.042 | 2.168 | 0.125 |
| Grass cover (%) | 0.028 | 1.768 | 0.181 |
| Annual cover (%) | 0.032 | 1.825 | 0.152 |
| Perennial cover (%) | -0.028 | 0.283 | 0.747 |
| Species richness | -0.023 | 0.405 | 0.669 |
| Average turnover | -0.0254 | 0.3653 | 0.696 |
