# Supplementary material for: Hotspots of Community Change: Temporal Dynamics Are Spatially Variable in Understory Plant Composition of a California Oak Woodland
Source: PLoS One. 2015 Jul 29;10(7):e0133501. doi: 10.1371/journal.pone.0133501 (PMC4519272; doi:10.1371/journal.pone.0133501)
Supplement: S3 Table — Relationships between explanatory variables aspect (quantified as the deviation from a northerly aspect) and topographic position, and treatment type (Burned and grazed, Grazed, or Grazing removed) applied to three watersheds. Explanatory variables are from 54 study plots distributed across the three watersheds. Values for χ2 statistics, degrees of freedom, and P-values are taken from a χ2 test quantifying the relationship between each variable and the treatment type. (DOCX) [file pone.0133501.s007.docx]

**S3 Table.**

| Variable | X square | df | P-value |
| --- | --- | --- | --- |
| Aspect | 3.156 | 2 | 0.206 |
| Topographic position | 12.824 | 8 | 0.118 |
